# Genomic and functional evaluation of exopolysaccharide produced by *Liquorilactobacillus mali* t6-52: technological implications

**DOI:** 10.1186/s12934-024-02431-z

**Published:** 2024-05-30

**Authors:** Manyu Wu, Shadi Pakroo, Chiara Nadai, Zeno Molinelli, Immacolata Speciale, Crisitina De Castro, Armin Tarrah, Jijin Yang, Alessio Giacomini, Viviana Corich

**Affiliations:** 1 Department of Agronomy Food Natural Resources Animal and Environment (DAFNAE), University of Padova, Padova, Italy; 2 Canadian Research Institute for Food Safety, Department of Food Science, University of Guelph, Guelph, ON N1G 2W1 Canada; 3 Interdepartmental Centre for Research in Viticulture and Enology (CIRVE), University of Padova, Conegliano, TV Italy; 4 Department of Agricultural Sciences, University of Napoli Federico II, Portici, NA Italy; 5 Department of Chemical Sciences, University of Padova, Padova, Italy; 6 Department of Land, Environment, Agriculture and Forestry (TESAF), University of Padova, Padova, Italy

**Keywords:** *Liquorilactobacillus mali*, Exopolysaccharide, Genomic, Cryoprotection, Antioxidant

## Abstract

**Background:**

This study explores the biosynthesis, characteristics, and functional properties of exopolysaccharide produced by the strain *Liquorilactobacillus mali* T6-52. The strain demonstrated significant EPS production with a non-ropy phenotype.

**Results:**

The genomic analysis unveiled genes associated with EPS biosynthesis, shedding light on the mechanism behind EPS production. These genes suggest a robust EPS production mechanism, providing insights into the strain’s adaptability and ecological niche. Chemical composition analysis identified the EPS as a homopolysaccharide primarily composed of glucose, confirming its dextran nature. Furthermore, it demonstrated notable functional properties, including antioxidant activity, fat absorption capacity, and emulsifying activity. Moreover, the EPS displayed promising cryoprotective activities, showing notable performance comparable to standard cryoprotective agents. The EPS concentration also demonstrated significant freeze-drying protective effects, presenting it as a potential alternative cryoprotectant for bacterial storage.

**Conclusions:**

The functional properties of *L. mali* T6-52 EPS reveal promising opportunities across various industrial domains. The strain’s safety profile, antioxidant prowess, and exceptional cryoprotective and freeze-drying characteristics position it as an asset in food processing and pharmaceuticals.

## Background

*Liquorilactobacillus mali* (formerly known as *Lactobacillus mali*) was initially identified by Carr and Davies [1]. It is one of the species within the *Liquorilactobacillus* genus and has been isolated from various sources, including fruit juice [2], cider [3], and water kefir grains [4, 5]. Despite limited research on *L. mali*, this bacterium has exhibited noteworthy functional and probiotic properties. Specifically, *L. mali* has been associated with the production of fermented sausages, contributing to their appealing sensory attributes and desirable textural qualities [6]. Additionally, the co-inoculation of yeast and *L. mali* has proven highly effective in promoting malolactic fermentation in the winemaking process [7]. Moreover, *L. mali* has shown probiotic potential due to its anti-obesity effects and role in treating non-alcoholic fatty liver disease by modulating specific gut microbiota in vivo [8].

Exopolysaccharides (EPS) are long-chain, high-molecular-weight, naturally produced biopolymers composed of sugar residues that microorganisms secrete into the surrounding environment [9]. EPS can be broadly classified into two main types based on their monosaccharide composition. The first category is homo-polysaccharides (HoPS), which exclusively consist of a single type of monosaccharide residue serving as the repeating unit. On the other hand, the second category is hetero-polysaccharides (HePS), characterized by the presence of two or more different monosaccharides in their structure. Examples of monosaccharides found in hetero-polysaccharides include mannose, galactose, and rhamnose [10]. EPS, being biodegradable, finds extensive applications in textiles, cosmetics, pharmaceuticals, bioremediation, food processing, and agriculture industries. Its eco-friendly nature makes it a versatile and valuable resource across diverse sectors [11]. As a natural food ingredient, EPS offers an alternative to artificial synthesis and chemical additives. Their interaction with food components contributes to thickening, emulsifying, gelling, and stabilizing food products [12]. Furthermore, it could enhance body texture, firmness, and creamy mouthfeel in food products [13].

Beyond their applications in food, EPS could play a crucial role in supporting the survival of probiotics during the gastrointestinal passage, facilitating their colonization in the human gut, and providing cryoprotection in processes such as freeze-drying [14]. Additionally, they serve as natural antioxidants, offering potential benefits in industrial and medical applications [15].

Recently, researchers worldwide have focused on extracting EPS from microorganisms due to its cryoprotective and freeze-drying protective activities. As a natural product, EPS offers competitive advantages over synthetic reagents such as dimethyl sulfoxide (DMSO) and glycerol [16].

In a study by Atallah et al. [17], the viability of probiotics in frozen yogurt was sustained, with counts ranging from 3 to 6.5 log10 CFU g − 1 even after 2 months of freezing. The role of EPS in this context is noteworthy, as it has been shown to effectively improve the water-holding capacity and alter the microstructure of fermented milk. These findings emphasize the multifaceted contributions of EPS in enhancing the quality, longevity, and nutritional aspects of frozen food products.

Our investigation explores the potential of EPS from *L. mali* T6-52, isolated from grape pomace. This study involves a detailed characterization of EPS, encompassing various analyses for bacterial strains genotyping, genome sequencing, EPS production, purification, and structural characterization. Additionally, we evaluate the antioxidant activity and functional properties of EPS, emphasizing its promising cryoprotective and freeze-drying protective capacities.

## Materials and methods

### Bacterial strains and RAPD-PCR analysis

Fifty-three isolates were sourced from the collection of the Interdepartmental Centre for Research in Viticulture and Enology (CIRVE), University of Padova, Italy. All isolates were derived from six-month-old grape pomace and previously identified through Amplified Ribosomal DNA Restriction Analysis (ARDRA) and 16 S sequencing, as documented by Rodas et al. [18]. These isolates were preserved in De Man-Rogosa (MRS)broth (Merck KGaA, Germany) supplemented with 25% (v/v) glycerol (Sigma-Aldrich) at − 80 °C. Before each experiment, the isolates were reactivated by culturing in sterile MRS broth and incubating at 30 °C for 48 h.

DNA extraction from all isolates followed the method outlined by Tarrah et al. [19]. In this procedure, 5 mL of activated bacterial cultures underwent two washes with a sterile PBS solution. Fifty microliters of lysis buffer (0.05 M NaOH + 0.25% SDS, Sigma-Aldrich) was added to each sample. The samples were vortexed for 2 min and then incubated in a thermal cycler (Bio-Rad Laboratories, Hercules, CA, USA) at 94 °C for 15 min. Subsequently, the solutions underwent centrifugation at 13,000×g for 10 min, and the supernatants were collected and further diluted 100 times in ultrapure sterile water. Next, strain discrimination among various isolates was conducted through Random Amplified Polymorphic DNA (RAPD) analysis. RAPD-PCR was performed using primer M13 (5′ GAGGGTGGCGGTTCT 3′) according to the protocol by Andrighetto et al. [20]. The electrophoretic profiles were analyzed using the software package Gel Compare Version 4.1 (Applied Maths, Kortrijk, Belgium) based on the Pearson product-moment correlation coefficient, and the dendrogram was obtained by using the Unweighted Pair Group Method with Arithmetic Mean (UPGMA).

### EPS production screening

The strains were cultured on modified MRS Agar medium supplemented with 10% sucrose (Sigma-Aldrich, USA). The plates were then incubated for 3–5 days at 30 °C. Identification of an EPS-positive strain was based on the observation that, upon touching the colonies grown on the plate with an inoculation loop, it produced a continuous thread, commonly referred to as the “ropy or mucoid character” [21, 22].

### Genome sequencing, assembly, and annotation

Genomic DNA from strain T6-52 was obtained using the DNeasy PowerSoil Kit according to the manufacturer’s instructions (Qiagen, Hilden, Germany). The quality of the DNA was checked using a Spark 10 M spectrophotometer (Spark 10 M, Tecan GmbH, Grödig, Austria). To sequence the genomes, we used paired-end sequencing with an Illumina MiSeq sequencer (BMR, Padua, Italy). The Unicycler assembler was used to assemble the raw reads with default settings [23], and the Rapid Annotation using Subsystems Technology (RAST) was employed for genome annotation [24]. To assess the safety of the strain, we examined the genome for the presence of virulence factors and acquired genes related to antibiotic resistance. The Comprehensive Antibiotic Resistance Database (CARD) server was employed to search for the presence of these genes, setting the server to perfect, strict, and loose hits. As a confirmatory step, we used the ResFinder 4.1 server to identify any acquired existing antibiotic-resistance genes [25, 26]. Finally, the predicted genome of T6-52 was investigated for EPS-related genes.

### EPS production, quantification, and purification

The EPS-producing strain *L. mali* T6-52 was incubated in modified MRS broth with 10% sucrose for 24 h at 37 °C. Subsequently, to remove impurities, trichloroacetic acid (TCA) was added to the culture to achieve a final concentration of 4% (w/v), and the mixture was stirred for 30 min at room temperature. To remove the cells and the precipitated proteins, the culture was centrifuged at 11,000 g for 10 min, and the supernatant was collected. Double-volume cold ethanol was added to the supernatant and incubated overnight at 4 ℃ to precipitate crude EPS. The precipitated EPS was collected by centrifuging at 2,500 g for 20 min. To remove small molecule impurities, the crude EPS was dissolved in distilled water and dialyzed using 12 kDa cellulose membrane (SERVA, Germany) against deionized water for 24 h with two times water change at room temperature. The purified EPS was lyophilized and used for further analysis. The EPS was weighed before and after purification separately [27–29].

### Total carbohydrate and protein concentration

The total carbohydrate content of EPS was determined by the colorimetric phenol-sulfuric acid method described by Dubois et al. [30]. The total protein concentration of EPS was determined by Bradford assay using Pierce™ Coomassie Plus (Bradford) Assay Kit (Thermo Fisher Scientific, USA).

### HPLC analysis

To analyze the carbohydrates composing the EPS, 10 mg of freeze-dried material was pre-treated according to Dallies et al. [31] with 100 µL of 72% H_2_SO_4_ for 3 h at 27 °C in vacuum-sealed tubes. Then, an acid solution was diluted to 2 N with distilled water up to a volume of 1200 µL. Tubes were then incubated at 100 °C for 4 h. 900 µL of liquid hydrolysate was collected, diluted, neutralized with HCl, and brought to a final volume of 10 mL. Analysis of monomers liberated from acid hydrolysis was performed upon PMP derivatization, according to Wang et al. [32]. Briefly, 150 µL of hydrolysate was mixed with 150 µL of aqueous 0.3 M NaOH and 150 µL of methanolic 0.5 M PMP solutions, then incubated for 90 min at 70 °C and subsequently cooled down. The mixture was then neutralized with 150 uL of aqueous 0.3 M HCl and three times washed with 500 µL of chloroform to eliminate unreacted excess of PMP. Derivatized samples were 0.2 μm filtered and run through a Polaris C18A column (4.6 × 250 mm, 5 μm, Agilent Technologies, Santa Clara, CA, USA) mounted on High-Performance Liquid Chromatography (HPLC) system composed by DGU-20 A 5R degasser, LC-20AD binary pump, SIL-20AC injector, CTO-20 A oven, SPD-M20A DAD detector (Shimadzu Co, Kyoto, Japan), using a mobile phase composed by (A) phosphate buffer 50 mM, pH 6.80, and (B) acetonitrile. The injection volume was 10 µL, the flow rate was 0.8 mL/min, and the oven temperature was 28 °C. On-line mixing conditions were 0–12 min B = 10%, 12–24 min B = 15%, 24–35 min B = 18%. Retention times for compound identification were obtained through runs of derivatized monosaccharide standards, as D-(+)-Mannose, D-(+)-Glucosamine hydrochloride, D-(+)-Glucose, L-Rhamnose, D-(+)-Xilose, and L-(+)-Arabinose, from Sigma. The detection wavelength was fixed at 245 nm.

### GC-MS analysis

The Gas Chromatography-Mass Spectrometry (GC-MS) linkage analysis was performed following Speciale et al. [33] to determine the linkages between sugar units in the EPS. All the samples were analyzed by GC–MS with an Agilent instrument (GC instrument Agilent 6850 coupled to MS Agilent 5973), equipped with a SPB-5 capillary column (Supelco, 30 m × 0.25 i.d., flow rate, 0.8 mL min − 1) and He as carrier gas. Electron impact mass spectra were recorded with an ionization energy of 70 eV and an ionizing current of 0.2 mA. The temperature program used for all the analysis was the following: 150 °C for 5 min, 150 → 280 °C at 3 °C/min, 300 °C for 5 min.

### NMR analysis

Nuclear Magnetic Resonance (NMR) analysis was carried out to provide detailed structural information about the EPS [34]. The NMR analyses were performed on a Bruker 600 MHz equipped with a cryogenic probe, and spectra were recorded at 298 K. Acetone was used as internal standard (1 H 2.225 ppm, 13 C 31.45 ppm) and 2D spectra (1 H–1 H DQF-COSY, 1 H–1 H NOESY, 1 H–1 H TOCSY, 1–13 C HSQC and 1–13 C HMBC) were acquired by using Bruker software (TopSpin 2.0). 31P NMR experiments were carried out with a Bruker DRX-400 spectrometer; aqueous 85% phosphoric acid was used as an external reference (δ = 0.00 ppm). Homonuclear experiments were recorded using 512 FIDs of 2048 complex with 32 scans per FID; mixing times of 100 and 200 ms were used for TOCSY and NOESY spectra acquisition, respectively. HSQC and HMBC spectra were acquired with 512 FIDs of 2048 complex points, accumulating 40 and 80 scans, respectively. Spectra were processed and analyzed using a Bruker TopSpin 3 program.

### Scanning electron microscopy (SEM)

The morphological features of the EPS sample were observed by using (Zeiss Supra VP35, Germany). One mg lyophilized EPS was dissolved in 1 mL Milli-Q water and then drop-cast on a glassy carbon substrate. The Scanning Electron Microscopy (SEM) micrographs were taken at ×600 × 1000 × 2000 magnifications and a voltage of 2 ~ 5 kV [35].

### Antioxidant activity

The total radical-scavenging capacity was determined by the DPPH (2-diphenyl-1-picryl-hydrazylhydrate) test, according to the method described by Hamidi et al. [36], with some modifications. The EPS solutions at five concentrations (0.25, 0.5, 1.0, 2.0, 4.0 mg/mL) were prepared using distilled water to quantify the antioxidant activity. Briefly, 1.0 mL of EPS solution (in sterilized distilled water) was mixed with 0.2 mL of a methanol solution of DPPH (0.1 mM) and 2.0 mL DH2O was added to the solution. The mixed solution was incubated in the dark at room temperature for 30 min. The absorbance of the EPS sample was measured at 517 nm using a UV-VIS spectrophotometer 55 (Ultrospec 2000, Pharmacia Biotech, England). The L-ascorbic acid (0.25 mg/mL) was used as a positive control. The radical scavenging ability (% of inhibition) was determined using the following formula:

$${\rm{Inhibition}}\,\left( {\rm{\% }} \right)\,{\rm{ = }}\,{\rm{1}} - \frac{{Ab{s_{sample}} - Ab{s_{blanksample}}}}{{Ab{s_{control}} - Ab{s_{blank}}}}) \times 100$$

### Fat absorption capacity

The fat absorption capacity of EPS was determined following the method of Guil-Guerrero et al. [37] with some modifications. A quantity of 0.1 g of EPS was suspended with 5 mL soybean oil in a falcon tube. The solution was vortexed 30 s every 5 min at room temperature. After 30 min, the mixture was centrifuged at 3500 × g for 25 min. The free oil was discarded, and the residual sample was weighed. The test was conducted three times independently. The Fat absorption capacity was calculated using the following formula:

$${\rm{Fat}}\,{\rm{absorption}}\,{\rm{capacity}}\,\left( {\rm{\% }} \right){\rm{ = }}\frac{{oil\,bound\,sample\,weight}}{{initial\,sample\,weight}} \times 100$$

### Water-solubility index and water-holding capacity

The water-solubility index (WSI) of EPS was measured following the method of Yang et al. [38] with slight modification. A quantity of 0.05 g of lyophilized EPS was suspended in 1 mL of deionized water and strongly vortexed for 2 h at room temperature. The mixture was centrifuged at 16,000 g for 25 min, and then the precipitation was freeze-dried again. The test was conducted three times independently.

The WSI was calculated according to the following equation:

$${\rm{WSI}}\,\left( {\rm{\% }} \right)\,{\rm{ = }}\frac{{m0 - m1}}{{m0}} \times 100$$

The m0 is the weight of initial lyophilized EPS; m1 is the weight of precipitated lyophilized EPS.

The water-holding capacity (WHC) of EPS was determined following Ahmed et al. [39]. A quantity of 0.2 g of EPS was suspended in 10 mL deionized water and then centrifuged at 16,000 × g for 25 min. The unbound water (not held by EPS) was discarded, and the surface moisture was wiped off with filter paper. The test was conducted three times independently. The WHC was calculated using the following formula:

$${\rm{WHC}}\,\left( {\rm{\% }} \right)\,{\rm{ = }}\frac{{total\,sample\,weight\,after\,water\,absorption}}{{total\,dry\,sample\,weight}} \times 100$$

### Emulsifying activity and emulsifying stability

The EPS emulsifying activity and emulsifying stability of oil were assessed using the method of Cooper and Goldenberg [40] and Prasanna et al. [41] with some modifications. An aliquot of 2 mL EPS solution (1%, w/v) was transferred into a falcon tube with 3 mL soybean oil; the mixture was vortexed on the cyclomixer for 2 min. The emulsion layer was determined and the emulsifying activity was calculated by the equation:

$${\rm{Emulsifying}}\,{\rm{activity}}\,\% \left( {{\rm{EA}}} \right)\,{\rm{ = }}\frac{{volume\,of\,the\,emulsion\,layer}}{{total\,solution\,volume}} \times 100$$

The emulsion layer was determined after 1, 12, 24 and 48 h. The emulsifying stability was calculated as:

$${\rm{Emulsifying}}\,{\rm{stability}}\,{\rm{\% }}\,\left( {{\rm{EA}}} \right)\,{\rm{ = }}\frac{{volume\,of\,the\,emulsion\,layer}}{{total\,solution\,volume}} \times 100$$

### Cryoprotective and freeze-drying shielding

The cryoprotective and freeze-drying shielding capabilities of EPS were assessed using the commercial probiotic *Lactiplantibacillus plantarum* 299 V as an indicator strain, following the method by Zhang et al. [16] with minor modifications. The strain was cultured in MRS broth at 37 ℃ for 18 h, and the resulting culture was centrifuged at 5,500 rpm for 5 min. After discarding the supernatant, the pellets were washed once and resuspended in an equal volume of fresh MRS broth. Following serial dilution, the bacterial concentration was determined by spreading an aliquot of the diluted bacterial suspension onto MRS plates, and the colony-forming units (CFU) were subsequently calculated.

For the cryoprotective activity assay, an aliquot of the bacterial suspension was added to cryoprotective solutions sterilized by autoclaving (121 ℃ for 15 min), containing 1.25%, 2.5%, and 5% (w/v) EPS. Control solutions comprising 20% (v/v) glycerol and 0.9% saline were also employed. The treated bacterial cells were stored at -80 ℃ for 3 weeks and 3 months.

In the freeze-drying protective activity assay, positive controls included 15% (w/v) sucrose and 10% (w/v) skim milk, while a 0.9% saline solution served as the negative control. The treated bacterial cells were similarly stored at -20 ℃ for 3 weeks and 3 months.

After storage, the survival rate was calculated as the percentage of culturable cell concentration post-storage relative to the cell concentration before storage (frozen or freeze-drying).

## Results

### RAPD-PCR and EPS screening

**Fig. 1:**
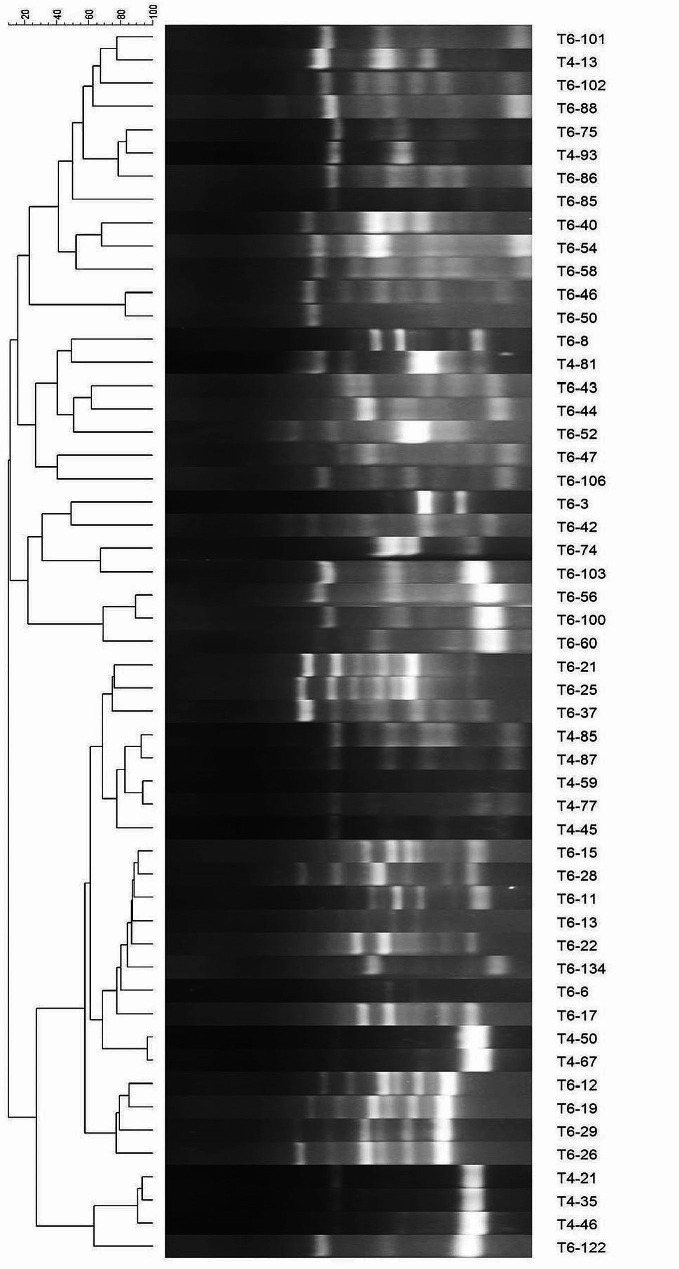
Cluster analysis of RAPD-PCR. The dendrogram illustrates the genetic relationships among 53 isolates based on RAPD-PCR fingerprints

Fifty-three isolates from the stock were subjected to RAPD-PCR analysis to discern their genetic distribution. The UPGMA analysis, performed by Gel Compare software, uncovered distinctive patterns within the isolate population. Following the analysis, 35 strains were identified as unique entities (Fig. 1). To ensure the reliability of subclusters, a minimum cophenetic correlation of 80% was applied, resulting in the selection of these 35 strains for subsequent tests. All discriminated strains underwent testing for EPS production using MRS agar supplemented with 10% sucrose. Among these, strains T6-15, T6-19, T6-26, and T6-60 exhibited the production of sticky filaments, measuring 3 mm, 7 mm, 4 mm, and 6 mm, respectively, indicative of a ropy phenotype. Notably, strain T6-52 demonstrated the highest EPS production and displayed a non-ropy phenotype.

### Genome sequencing, assembly, and annotation

**Fig. 2:**
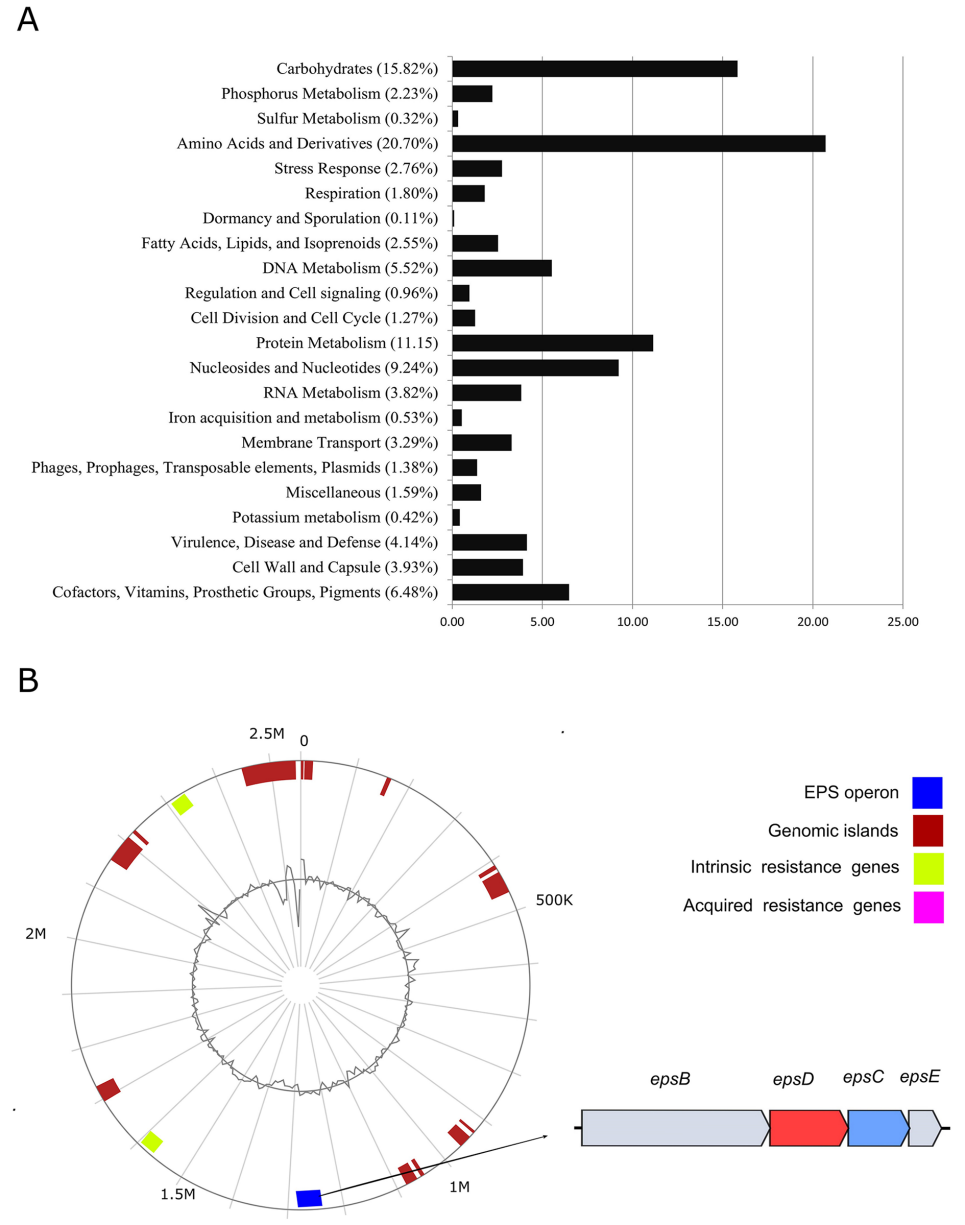
(**A**) The figure provides an overview of subsystems within the sequenced genome, highlighting key functional categories. (**B**) The circular representation visualizes the genomic landscape, with specific emphasis on the *eps* operon

The genome that was assembled consisted of 44 contigs grouped into 20 scaffolds, resulting in a genome size of 2.53 Mb and a GC content of 36.5%. Annotation via the RAST server revealed 2558 anticipated protein-coding sequences (CDSs) sorted into 226 SEED subsystems. Additionally, 48 structural RNAs and 26 genes associated with various stress reactions (such as osmotic, oxidative, and detoxification) were identified after annotation. The most significant portion of the T6-52 subsystem pertains to amino acids and derivatives followed by carbohydrate metabolism. Within carbohydrate metabolism, monosaccharides possess the most genes, followed in descending order by core carbohydrate metabolism, di- and oligosaccharides, fermentation, amino sugars, sugar alcohols, organic acids, and finally, one-carbon metabolism (Fig. 2A).

Genomic analysis revealed the absence of virulence genes, and no acquired antibiotic resistance genes were detected. Only intrinsic resistance related to fluoroquinolone and beta-lactamase was identified, which poses no risk to consumers (Fig. 2B).

The genome contains 20 genes associated with capsular and extracellular polysaccharides. Out of these 20 genes, 6 are related to dTDP-rhamnose synthesis, 9 pertain to rhamnose-containing glycans, and 5 are linked to EPS biosynthesis. Specifically, we identified gene related to dextransucrase involved in homopolysaccharide polymerization. Upon delving deeper into the EPS biosynthesis, we identified genes associated with the EPS operon: *epsB*, *epsC*, *epsD*, and *epsE* genes (Fig. 2B).

### EPS production and composition

Following the purification process, the obtained EPS demonstrated a notable concentration, registering at 660 ± 16 mg/L. A comprehensive compositional analysis unveiled the richness of the EPS, with a predominant total carbohydrate content of 81.18%. Additionally, a minimal yet significant presence of total protein was estimated at 0.12%, emphasizing the intricate nature of the purified exopolysaccharide.

### Structural analysis of EPS

**Fig. 3:**
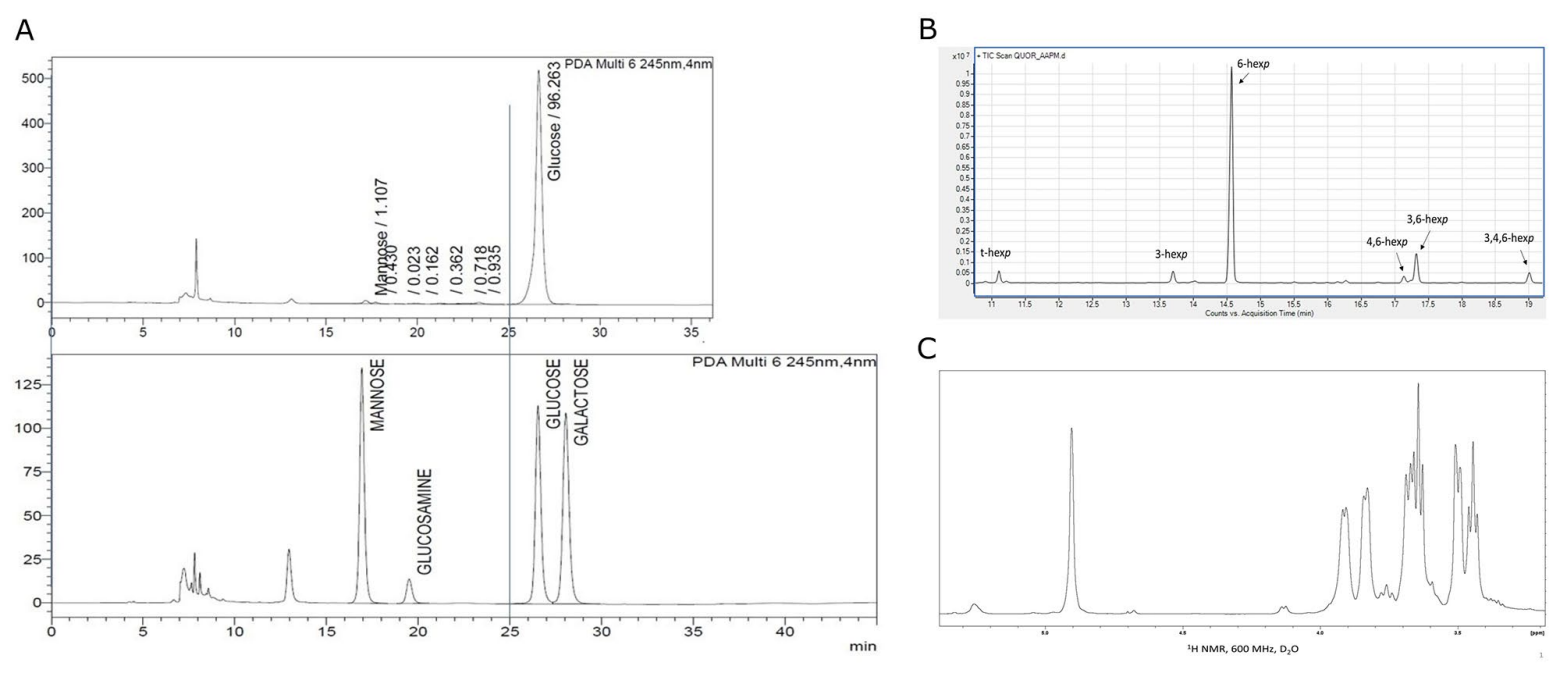
Analysis of EPS structures. (**A**) HPLC: Composition overview (Upper: Composition of analyzed EPS. Lower: HPLC chromatogram corresponding to the reference used for comparison.), (**B**) GC-MS: Linkage analysis, and (**C**) 1 H NMR: Structural details

To unveil the structure of EPS, we conducted HPLC-DAD analysis after EPS underwent acid hydrolysis. The resulting chromatogram distinctly revealed the presence of a single monosaccharide, constituting the 96.26% of the total chromatogram area, unequivocally identified as glucose based on retention time (Fig. 3A). This analytical information indicated that the EPS from *L. mali* T6-52 is a homopolysaccharide composed of glucose.

GC–MS analysis further corroborated these findings, confirming that the predominant monosaccharide in *L. mali* T6-52 EPS is indeed glucose. Noteworthy, methylation analysis, carried out to establish the glucose attachment point [42], showed the presence of 6-substituted glucopyranose (6-hex*p*), as the prevalent sugar in the polysaccharide (Fig. 3B).

Delving deeper into the molecular structure, we have run ^1^H NMR spectroscopy by which it was evident that the EPS possesses a dextran structure (Fig. 3C) [43]. Intriguingly, in this spectrum were present monior anomeric proton signals responsible of the dextran branching points as also shown in the methylation analysis. Collectively, these analyses unequivocally establish *L. mali* T6-52 EPS as a homopolysaccharide, classified as dextran.

### SEM analysis

**Fig. 4:**
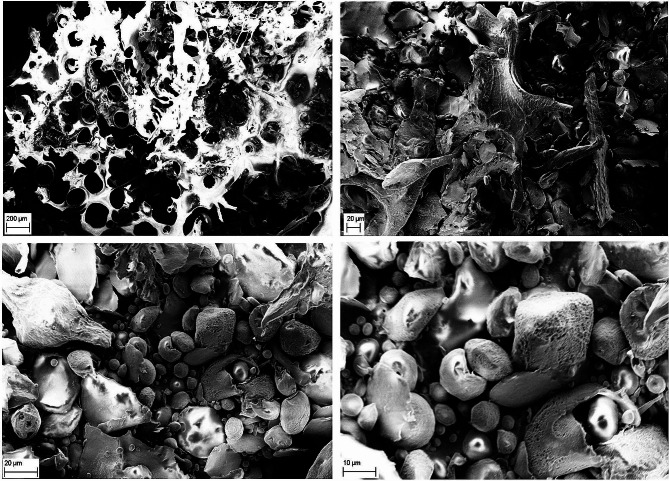
The figure presents the morphology of EPS under Scanning Electron Microscopy (SEM) using various magnifications (×100, ×600, ×1500, and ×3000)

The SEM analysis provided a detailed insight into the macrostructure and microstructure of the purified lyophilized EPS (Fig. 4). The EPS, synthesized by *L. mali* T6-52, exhibited a distinctive sheet-like appearance, showcasing a flat and extended structure. Under varying magnifications (×100, ×600, ×1500, and ×3000), the micrographs revealed a smooth surface, indicating the absence of irregularities. Furthermore, the tight structures observed within each segment emphasized a closely packed arrangement, with discernible interstices contributing to the overall intricate architecture of the EPS.

### Antioxidant activity

**Fig. 5:**
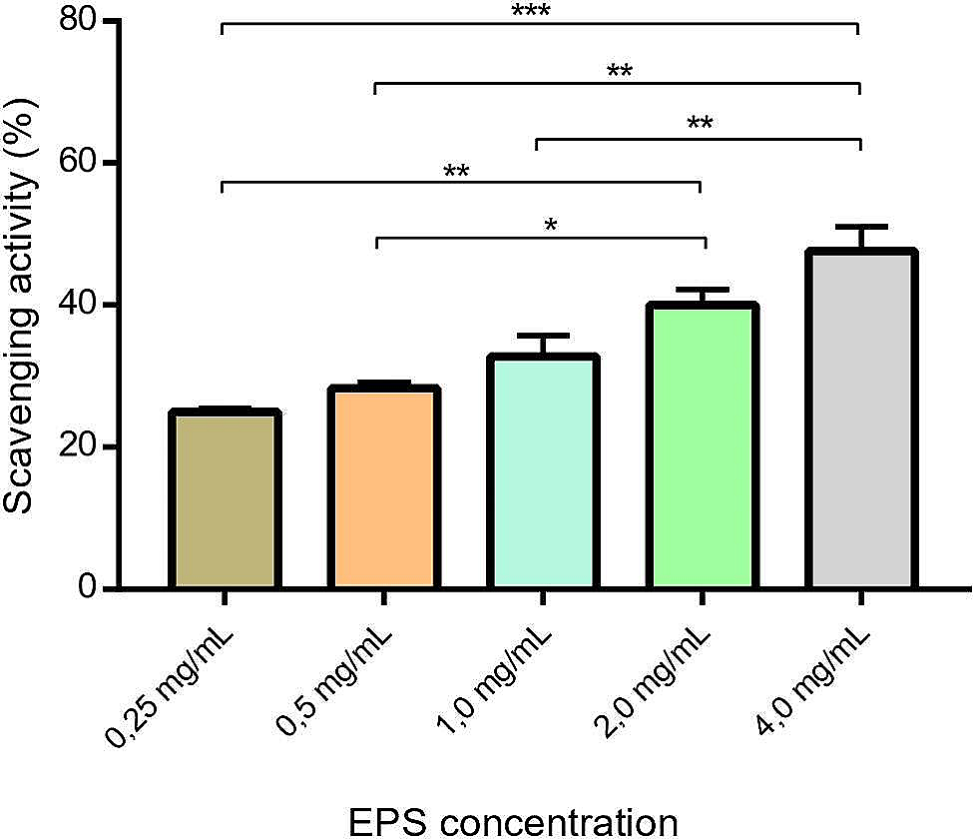
Antioxidant activity of different concentrations of EPS

The assessment of DPPH radical scavenging activity involved the examination of various concentrations of purified EPS and ascorbic acid (ranging from 0.25 to 4.0 mg/mL), as illustrated in Fig. 5. The observed values indicated a discernible trend wherein the scavenging activity of the EPS exhibited a consistent increase with ascending concentrations, ranging from 24.93% at the lowest concentration to 47.62% at the highest concentration. This observed concentration-dependent enhancement in radical scavenging underscores the potential antioxidant properties of the examined EPS, providing valuable insights into its efficacy across varying concentrations. Notably, ascorbic acid, used as a positive control, displayed significant antioxidant activity of 92%.

### Functional properties

In our investigation of functional properties, the fat absorption capacity of the EPS emerged prominently, showcasing 621.00 ± 12.25%. Methodical determination of the WSI and WHC of EPS yielded values of 99.0 ± 0.7% and 139.33 ± 3.2%, respectively. The EPS exhibited EA, particularly concerning soybean oil. At a concentration of 1% (w/v), the EA for soybean oil surged to 92 ± 1.5%. Further analysis unveiled dynamic ES over various time intervals, with ES1h, ES12h, ES24h, and ES48h values standing at 54.35 ± 0.8%, 54.35 ± 0.9%, 52.17 ± 1.1%, and 52.17 ± 1.3%, respectively. These findings underscore the diverse functional attributes of *L. mali* T6-52 EPS, with significant implications for its potential applications across various food and industrial contexts.

### Cryoprotective and freeze-drying protective activities

**Fig. 6:**
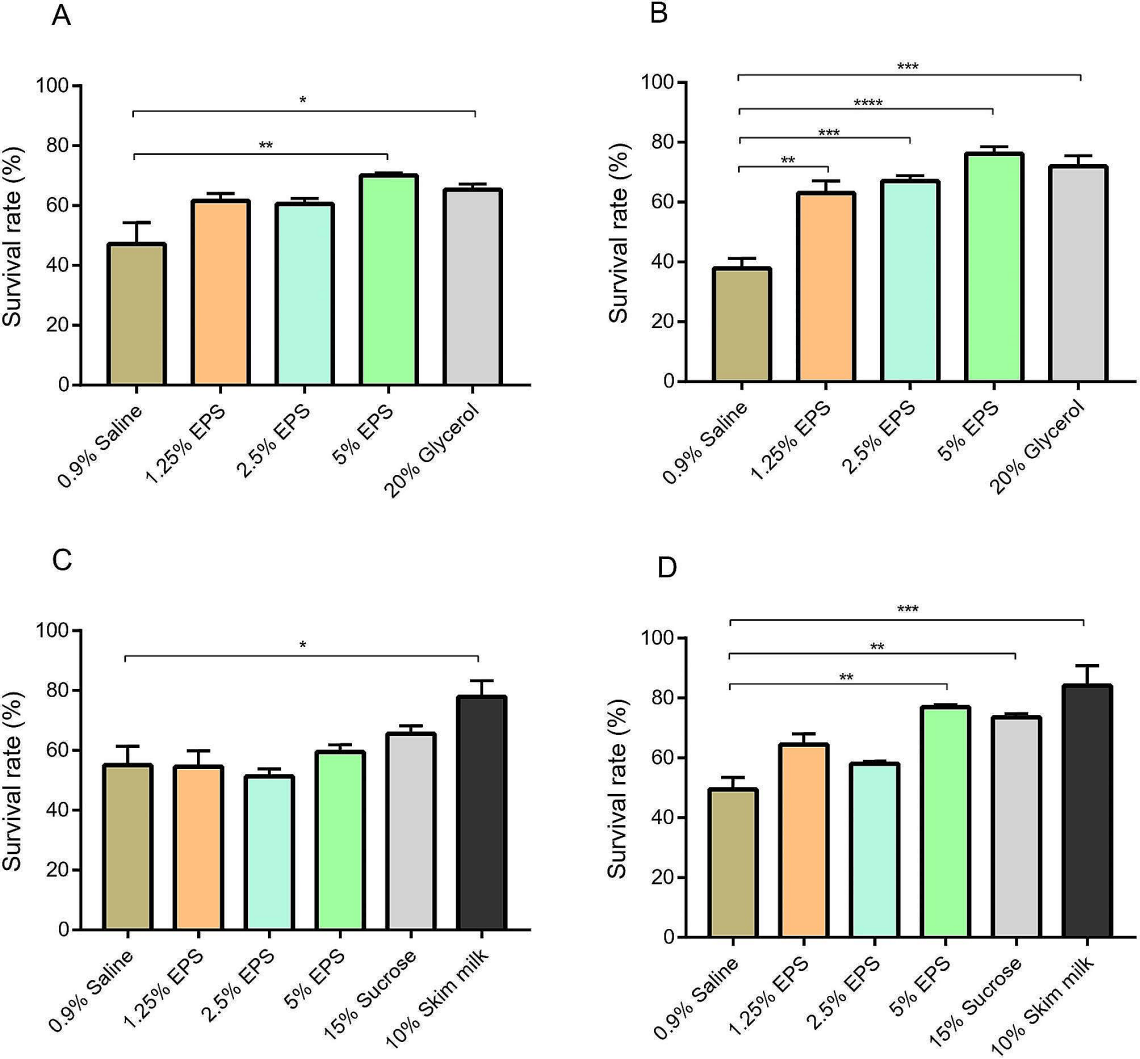
**A**) Cryoprotective effects of EPS after 3 weeks. **B**): Cryoprotective effects of EPS after 3 months. **C**) Freeze-drying shielding effects of EPS after 3 weeks. **D**) Freeze-drying shielding effects of EPS after 3 months

The investigation into the cryoprotective and freeze-drying protective activities of EPS yielded insightful results. Following a rigorous storage regimen at -80 ℃ for 3 weeks and 3 months, the survival rates of *L. plantarum* 299v were determined, with outcomes graphically presented in Fig. 6A-B. The cryoprotective efficacy of EPS was compared with 0.9% saline solution (negative control) and 20% glycerol (positive control). Notably, the survival rates of cells preserved in varying EPS concentrations, namely 1.25%, 2.5%, and 5%, surpassed those in the saline solution after 3 months of incubation, showcasing the pronounced cryoprotective effects of EPS. Impressively, all EPS concentrations exhibited protection comparable to the 20% glycerol control after 3 months. Furthermore, during the 3-week incubation period, notable protective effects were observed, specifically with the 5% EPS concentration. This suggests a noteworthy trend wherein the cryoprotective effectiveness of EPS becomes more pronounced with extended incubation periods at -80 ℃, underscoring the dynamic nature of its protective capabilities over time.

Continuing the investigation, lyophilization and subsequent storage for 3 weeks and 3 months provided insights into the freeze-drying protective activities of EPS, as delineated in Fig. 6C-D. A comprehensive comparison with 0.9% saline solution (negative control) and positive controls, 15% sucrose and 10% skim milk, further underscored the efficacy of EPS in preserving *L. plantarum* 299 V. Intriguingly, during the initial 3-week incubation, only skim milk, the positive control, indicated significant protection. However, after the extended 3-month storage period, the 5% EPS concentration exhibited protection comparable with the positive controls, emphasizing the remarkable freeze-drying protective capabilities of EPS.

## Discussion

Exopolysaccharides, intricate polymers secreted by microorganisms, play a pivotal role in various biological processes and industrial applications. The strain T6-52, characterized by a non-ropy phenotype, demonstrated a good capacity for EPS production, exhibiting properties such as high surface hydrophobicity and free sulfhydryl content, resulting in enhanced hardness and reduced water holding capacity [44]. Zhang et al. emphasize the importance of such characteristics in improving the crispiness of food products while minimizing the risk of rupture [44]. The non-ropy EPS also displayed higher shear resistance, suggesting improved cohesion and reduced flow tendency, making it suitable for incorporation into solid fermented food products [45]. Nachtigall et al. [46] also reported similar characteristics, noting higher moisture load capacity and slower moisture release in fermented milk products. These findings collectively position non-ropy EPS as an excellent candidate for enhancing fermented flour and dairy products.

Genomic analysis in our study revealed the absence of virulence genes and the lack of acquired antibiotic resistance genes, indicating intrinsic resistance related to fluoroquinolone and beta-lactamase. This intrinsic resistance enhances the strain’s survivability and persistence in environments where antibiotics may be present [47]. Moreover, our genomic analysis unveiled several genes associated with EPS biosynthesis, which are linked to the biosynthetic pathway of EPS production, known as the dextran biosynthetic pathway. Specifically, we identified gene related to dextransucrase involved in homopolysaccharide polymerization.  The genes *epsB* and *epsE* were detected within the EPS operon, suggesting their roles in coordinating EPS biosynthesis and export processes. The *epsB* gene is involved in the early stages of EPS synthesis, coordinating the formation of vital precursor molecules and the gene *epsE* is intricately linked to the terminal stages of EPS synthesis or even the export mechanisms, ensuring that the synthesized EPS efficiently navigates the dense cell walls characteristic of many LAB species and reaches its functional destination in the extracellular environment [49]. These findings align with the chemical characterization of the EPS as a homopolysaccharide composed primarily of glucose, as elucidated through analytical techniques. Moreover, methylation analysis identified 6-substituted glucopyranose as the prevalent sugar in the EPS, providing insights into the biosynthetic pathway. The integration of genomic data with chemical structure elucidation establishes a direct correlation between the identified genes and the biosynthesis of EPS in *L. mali* T6-52, enhancing our understanding of EPS production at the genetic level. LAB are renowned for their essential role in food fermentation and have attracted significant attention due to their ability to produce EPS. The synthesis and export of EPS in LAB can influence the texture and sensory characteristics of fermented foods, as well as potential health implications [12, 13].

Furthermore, EPS biosynthesis plays a critical role in various cellular processes, from adherence to surfaces to biofilm formation, providing protective barriers, and interactions with the host [48].

The comprehensive chemical composition analysis identified the EPS as a homopolysaccharide, primarily composed of glucose and recognized as dextran. This homogeneity in structure and composition holds significant promise for various applications. In particular, the homogeneity in term of chemical structure of the homo EPS offers consistency in properties, making it well-suited for applications demanding stable and predictable performance. Its glucose-based dextran nature opens avenues for specific functionalities, positioning it favorably in the food industry as an additive, stabilizer, or emulsifier [50]. The compatibility of homo EPS with diverse industrial processes enhances its potential integration into various formulations. Beyond the food sector, the homogenous structure may find applications in biotechnological fields, where precision in polymer properties is paramount [51].

The antioxidant activity exhibited by *L. mali* T6-52 EPS adds a distinctive dimension to its functional properties. The EPS scavenging activity exhibited a proportional increase with rising EPS concentration, ranging from 24.93 to 47.62%. This aligns with findings by Adesulu-Dahunsi et al. [52], who reported a comparable range of EPS scavenging activity (19.8–48.9%) across concentrations from 0.5 to 4 mg/mL. In addition, Zhang et al. [53] noted that, at a 4.0 mg/mL concentration, EPS demonstrated 52.23% DPPH radical scavenging activity, with no further increase observed at higher concentrations.

Moreover, *L. mali* T6-52 EPS exhibited relatively good WSI and WHC. Comparing these activities with homopolysaccharides from LAB species reported in the literature reveals intriguing differences. For instance, when compared to *Leuconostoc pseudomesenteroide* XG5, T6-52 EPS exhibits higher WSI (99.0% vs. 90.2%) and lower WHC (139.33% vs. 412%) [54]. On the other hand, the WSI of a *Leuconostoc lactis* EPS (91.90 ± 2.45%) was close to *L. mali*, although the WHC was lower (509.45 ± 28.59%) [55]. Researchers note that these variations do not solely depend on monosaccharide composition but are influenced by factors such as hydrogen bond content and pore structure of EPS. The diverse WSI and WHC values among species impact the water absorptive ability, thus affecting the quality of food products. While high WSI and WHC maintain meat products juicy and fresh, low values enhance the crispiness and workability of extruded food products. EPS with high WSI and WHC can serve as potential water-binding and stabilizing agents, suggesting broad applications in the food industry [47, 51, 53, 54].

*L. mali* T6-52 EPS indicated EA and ES in soybean oil at a 1% concentration. Despite its low protein content (0.12%), the EPS revealed high activity and stability, highlighting the crucial role of factors such as the glycosidic chain, monosaccharide composition, and molecular weight. The stable emulsion formation suggests the potential for EPS in developing novel bio emulsifiers for various industries, including food, cosmetics, and pharmaceuticals [55–59].

The multifaceted functional profile of *L. mali* T6-52 EPS extends beyond its applications in food systems, encompassing cryoprotective and freeze-drying activities with notable industrial implications. The cryoprotective efficacy of the EPS positions it as a compelling alternative for bacterial storage [60]. In a study, EPS exhibited cryoprotection in frozen dough and frozen yogurt, two staples with an average storage duration of approximately three months. Terpou et al. [61] indicated high survival rates during three months of frozen storage, emphasizing the potential health benefits of probiotic-infused foods for consumers. Investigations into the role of LAB-derived EPS in frozen dough systems have revealed significant advancements. The study by Ouyang et al. [62] demonstrated that LAB-derived EPS plays a crucial role in forming a more compact glucan structure. This prevents internal system damage to the dough and enhances the baking quality of frozen dough bread, marking a pivotal development in frozen food technology. In addition, our study findings reveal a direct correlation between EPS yield and its protective properties. Our experimental results indicate that increasing EPS yield leads to enhanced cryoprotective and antioxidant activities. This suggests that variations in EPS yield directly impact its cryoprotective and antioxidant abilities, highlighting the potential of EPS as a multifunctional ingredient for improving the stability and quality of food products. This correlation underscores the importance of optimizing EPS production to maximize its protective effects in various applications, including food preservation and biotechnology.

In our study, the efficacy of EPS became more pronounced after an extended 3-month storage period. This intriguing phenomenon could be attributed to several factors, including the maturation of the EPS matrix, gradual interaction with bacterial cells, and concentration-dependent effects [60–63]. The extended storage period likely allows for the optimization of the EPS matrix, leading to a more effective shield against freeze-thaw stresses. The time-dependent improvement in cryoprotection aligns with the dynamic nature of EPS-mediated effects, suggesting that the full potential of EPS in preserving probiotics becomes more evident over extended storage durations.

The cryoprotective nature of the EPS also aligns with its other functional properties, creating synergies that could be used in various applications. For instance, the EPS’s antioxidant activity may contribute to preserving bacterial viability during freezing and thawing processes, further enhancing its role as a cryoprotectant [64]. Antioxidants are known to lower the freezing point of solutions, preventing ice crystal formation during freezing and storage. In the context of bacterial preservation, this property becomes crucial. The antioxidant capacity exhibited by *L. mali* T6-52 EPS may contribute to reducing the cryoscopic point of the preservation, minimizing ice crystal formation during freezing and thawing. This, in turn, enhances the cryoprotective potential of EPS, providing a mechanism for preserving bacterial viability during storage [65]. These findings suggest a dual functionality for *L. mali* T6-52 EPS, where its antioxidant activity contributes to potential health benefits and enhances its effectiveness as a cryoprotectant in various applications.

Integrating cryoprotection with other functional attributes presents a holistic approach to utilizing *L. mali* T6-52 EPS in industrial settings. The ability to simultaneously protect bacterial cells and contribute to the stability and quality of bioactive compounds opens avenues for streamlined processes in biotechnology and pharmaceuticals. The potential synergies between cryoprotection and other functional properties position *L. mali* T6-52 EPS as a versatile and valuable resource, showcasing its relevance beyond traditional applications in the food industry.

## Conclusion

In conclusion, the multifaceted capabilities of *L. mali* T6-52 EPS, spanning from its unique non-ropy phenotype and genomic insights to its chemical composition and functional properties, open avenues for diverse industrial applications. The strain’s safety profile and its antioxidant activity, cryoprotective, and freeze-drying properties suggest promising potential in food processing, pharmaceuticals, and beyond. The findings presented here contribute to understanding a robust EPS-producing bacterial strain and pave the way for future research exploring its practical utilization.

## Data Availability

No datasets were generated or analysed during the current study.
